# A survival case of visceral disseminated varicella zoster virus infection in a patient with systemic lupus erythematosus

**DOI:** 10.1186/s12882-023-03223-0

**Published:** 2023-06-08

**Authors:** Yuri Ishino, Hirotaka Fukasawa, Shuhei Kitamoto, Daisuke Nakagami, Mai Kaneko, Hideo Yasuda, Ryuichi Furuya

**Affiliations:** 1 Renal Division, Department of Internal Medicine, Iwata City Hospital, 512-3 Ohkubo, Iwata, Shizuoka 438-8550 Japan; 2 First Department of Medicine, Hamamatsu University School of Medicine, Hamamatsu, Shizuoka Japan

**Keywords:** Abdominal pain, Mycophenolate mofetil (MMF), Skin blisters, Systemic lupus erythematosus (SLE), Varicella zoster virus

## Abstract

**Background:**

Visceral disseminated varicella zoster virus (VZV) infection is a rare but life-threatening complication in immunosuppressed patients. Herein, we report a survival case of visceral disseminated VZV infection in a patient with systemic lupus erythematosus (SLE).

**Case presentation:**

A 37-year-old woman was diagnosed as SLE and initial induction therapy was started. Two months after starting the immunosuppressive therapy consisting of 40 mg of prednisolone (PSL) and 1500 mg of mycophenolate mofetil (MMF) daily, she suddenly developed strong abdominal pain, which was required opioid analgesics, followed by systemic skin blisters, which were diagnosed as varicella. Laboratory findings showed rapid exacerbation of severe liver failure, coagulation abnormalities and increased numbers of blood VZV deoxyribonucleic acid (DNA). Therefore, she was diagnosed as visceral disseminated VZV infection. Multidisciplinary treatment with acyclovir, immunoglobulin and antibiotics was started, the dose of PSL was reduced, and MMF was withdrawn. By their treatment, her symptoms were resolved and she finally discharged.

**Conclusions:**

Our case highlights the importance of a clinical suspicion of visceral disseminated VZV infections, and the necessity of immediate administration of acyclovir and reduced doses of immunosuppressant to save patients with SLE.

## Background

Varicella zoster virus (VZV) infections including varicella, herpes zoster and visceral disseminated VZV infection are often experienced in immunocompromised patients [1–3]. Among them, visceral disseminated VZV infection is the most serious complication with a high mortality rate in patients undergoing immunosuppressive therapy for kidney transplant recipients or hematological diseases [2, 4–7]. Recently, Habuka et al*.* [8] and Vassia et al*.* [9] reported the fatal cases of visceral disseminated VZV infection in patients with systemic lupus erythematosus (SLE) undergoing immunosuppressive therapy. Therefore, earlier diagnosis and medical intervention for this serious complication are required.

Herein, we report a case of visceral disseminated VZV infection, which developed 2 months after starting immunosuppressive therapy consisting of prednisolone (PSL) and mycophenolate mofetil (MMF) for SLE. The patient’s condition rapidly became serious, but resolved by prompt diagnosis and treatment.

## Case presentation

A 37-year-old Japanese woman was referred to our hospital for the evaluation of pancytopenia, hypocomplementemia, positivity of anti-nuclear antibody and proteinuria.

Physical examination on admission revealed height of 155 cm, body weight of 48.0 kg, body temperature of 39.1 ºC, pulse rate of 100 beats/min and blood pressure of 161/112 mmHg. There was no previous history of herpes zoster and were no symptoms including muscle weakness, difficulty of swallowing, shortness of breath, thickening of skin, and dry eyes or skin.

**Fig. 1:**
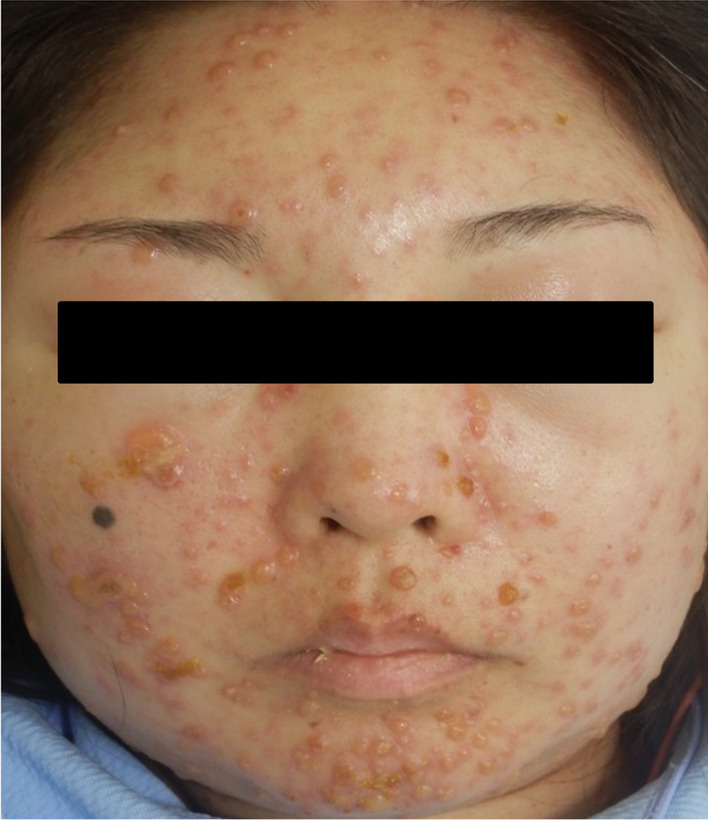
Finding of skin lesions on the face

**Fig. 2:**
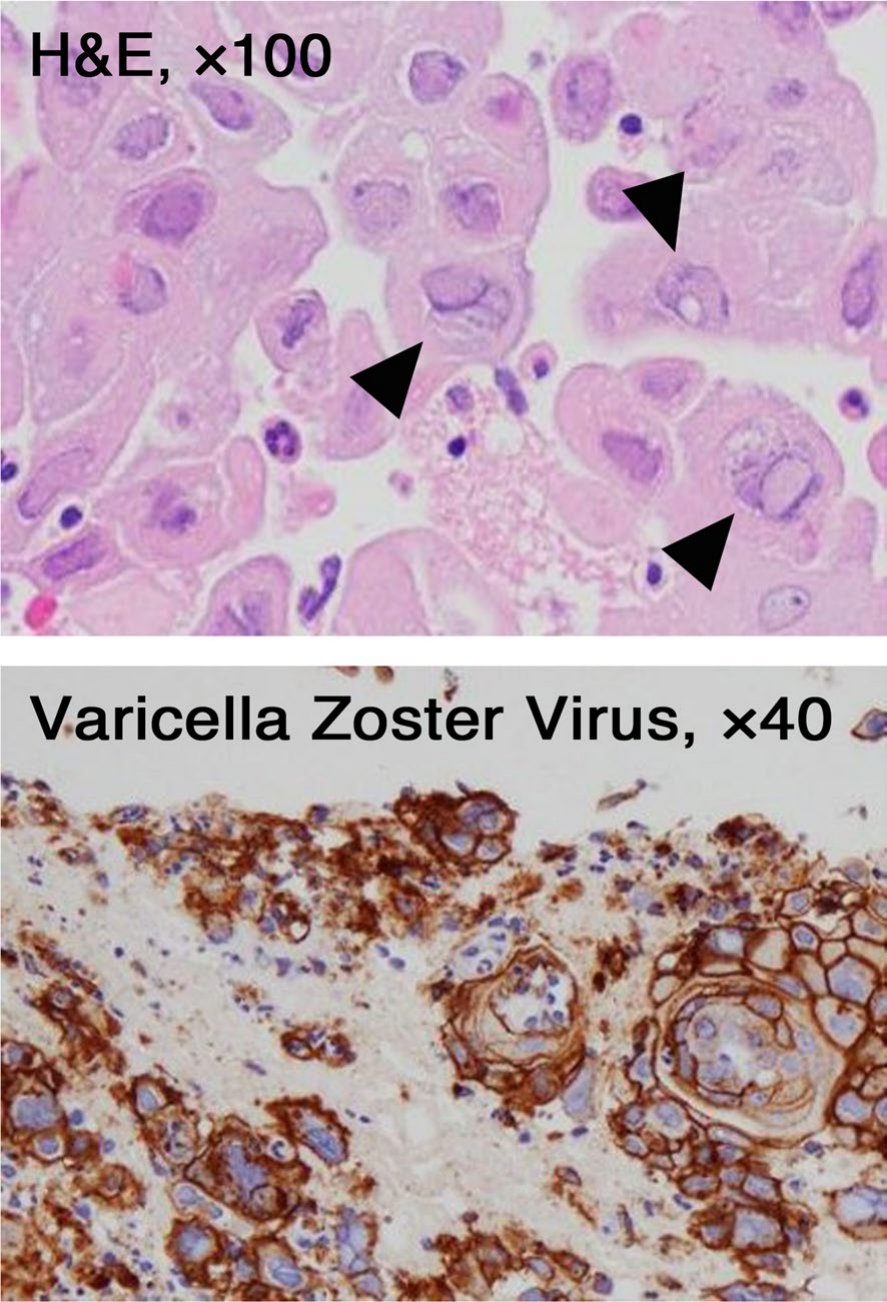
Findings of the biopsy specimen in the esophagus. (Upper panel) Hematoxylin and eosin (H&E) staining of the biopsy specimen in the esophagus (× 100). Inclusion bodies in nucleus, suggesting the presence of viral infection, were observed (arrow heads). (Lower panel) The staining with the antibody against VZV protein was also positive (× 40). Abbreviations: H&E, hematoxylin and eosin; VZV, varicella zoster virus

**Fig. 3:**
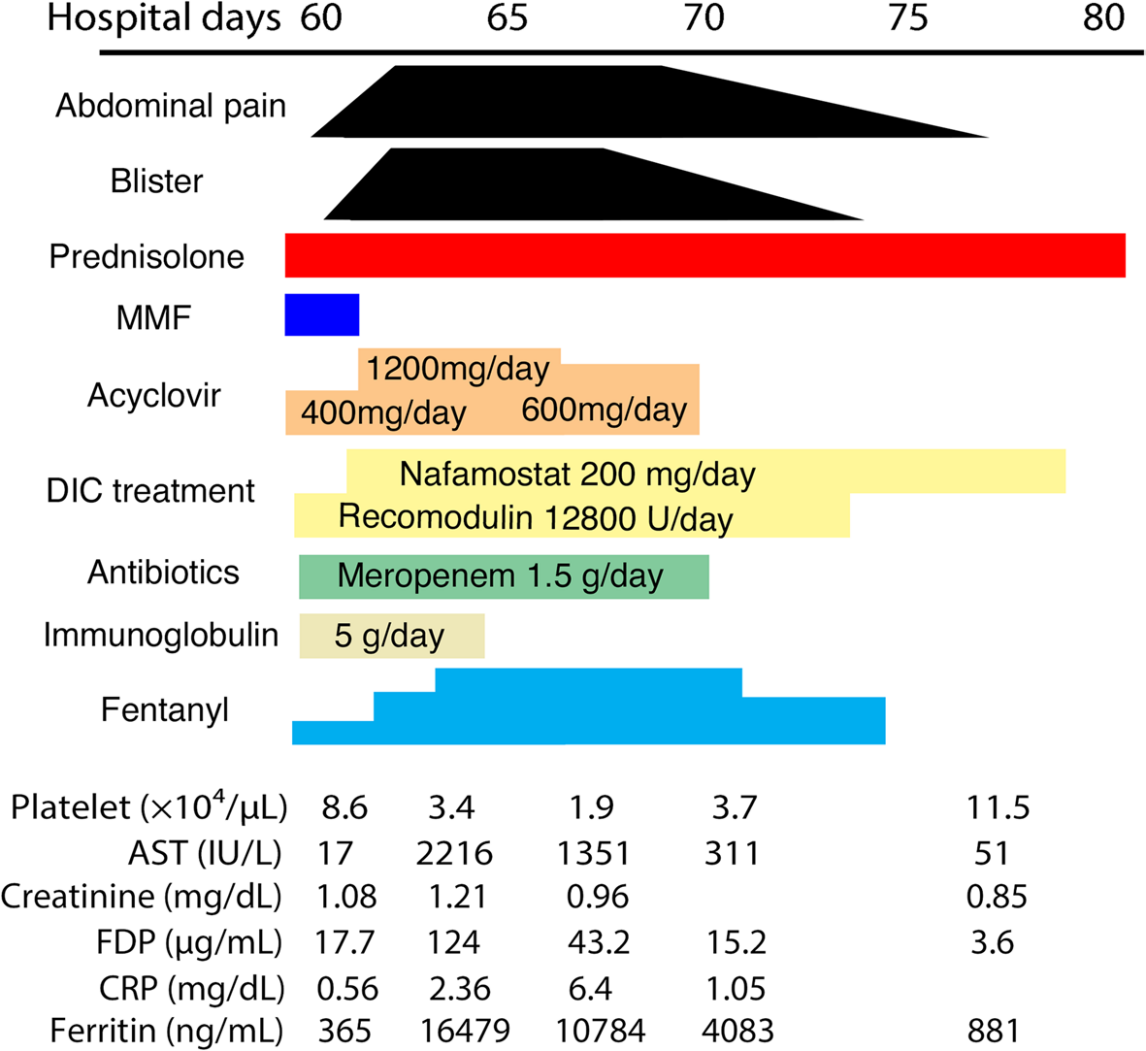
Clinical course after the onset of visceral disseminated VZV infection. Abbreviations: AST, Aspartate aminotransferase; CRP, C-reactive protein; DIC, Disseminated intravascular coagulation; FDP, Fibrinogen/fibrin degradation products; MMF, Mycophenolate mofetil; VZV, Varicella zoster virus

Laboratory studies on admission revealed a white blood cell count of 2,600 /mm^3^, hemoglobin of 7.7 g/dl, platelet count of 3.5 × 10^4^ /mm^3^, serum creatinine of 1.45 mg/dL, estimated glomerular filtration rate (eGFR) of 34 mL/min/1.73 m^2^, hypocomplementemia, positivity of anti-nuclear antibody and anti-deoxyribonucleic acid (DNA) antibody, proteinuria and hematuria (Table 1). In cardiac ultrasonography, bilateral pleural effusion and pericardial effusion were observed. Base on the criterion for the classification of SLE by American College of Rheumatology (ACR) [10] and Systemic Lupus International Collaborating Clinics (SLICC) [11], she was diagnosed as SLE. The Safety of Estrogens in Lupus Erythematosus National Assessment-Systemic Lupus Erythematosus Disease Activity Index (SELENA-SLEDAI) score [12] was 33. The initial induction therapy with 40 mg of prednisolone (PSL) daily was started without hydroxychloroquine. One month after starting PSL, 1500 mg of mycophenolate mofetil (MMF) daily was added. Two months after starting the induction therapy consisting of PSL and MMF, she suddenly developed abdominal pain. Upper gastrointestinal endoscopy and computed tomography (CT) scan were performed, although only mild erosion in esophagus and stomach was present, and there was no specific finding in CT scan. Three days after the onset of abdominal pain, systemic rash with small and fluid-filled blisters developed (Fig. 1), and she was diagnosed as varicella by the result of VZV antigen test (Dermaquick VZV, Maruho, Osaka, Japan) of vesicular fluid. So, we started the administration of acyclovir. On the same day, her abdominal pain worsened and required opioid analgesics. Laboratory findings showed rapid increases of serum liver enzymes, serum ferritin levels, coagulation abnormalities. Furthermore, increased copies of VZV DNA were detected in her blood. Viral inclusion body was also observed and the staining with the antibody against VZV protein (MAB8612, EMD Millipore Corporation, Temecula, USA) was positive in the specimen of esophageal erosion obtained from upper gastrointestinal endoscopy (Fig. 2). Therefore, she was diagnosed as visceral disseminated VZV infection. In addition to the administration of acyclovir, the treatment with antibiotics (meropenem 0.5 g × 3 daily) and immunoglobulin (5 g daily) was immediately and empirically started, the dose of PSL was reduced to from 40 to 30 mg daily, and MMF was withdrawn. Her general condition became serious, but her abdominal pain and skin blisters were subsequently resolved (Fig. 3). Finally, she discharged 3 months after the onset of VZV infection without the recurrence of SLE (SELENA-SLEDAI score was 4).

**Table 1:** Laboratory findings on admission

White blood cell	2,600 /mm^3^	CRP	0.12 mg/dL
Lymphocyte	670 /mm^3^	IgG	1263 mg/dL
Red blood cell	271 × 10^4^ /mm^3^	IgA	83 mg/dL
Hemoglobin	7.7 g/dL	IgM	55 mg/dL
Platelet	3,5 × 10^4^ /mm^3^	C3	19 mg/dL
		C4	3 mg/dL
Total protein	5.1 g/dL	CH50	< 12 U/mL
Albumin	2.4 g/dL	Anti-nuclear antibody	× 160, speckled
ALT	29 IU/L	Anti-dsDNA antibody	> 200 IU/mL
AST	16 IU/L	Anti-Smith antibody	< 1.0 U/mL
LDH	541 U/L	MPO-ANCA	< 1.0 U/mL
BUN	25 mg/dL	PR3-ANCA	< 1.0 U/mL
Creatinine	1.45 mg/dL		
eGFR	34 mL/min/1.73m^2^	Urinalysis	
Uric acid	8.8 mg/dL	Protein	(4 +)
Na	133 mEq/L	Occult blood	(3 +)
K	3.9 mEq/L	Urinary sediment	
Cl	106 mEq/L	Red blood cell	20–29 /HPF
Ferritin	152 ng/mL	White blood cell	1–4 /HPF

*Abbreviations*: *ALT* Alanine aminotransferase, *AST* Aspartate aminotransferase, *BUN* Blood urea nitrogen, *CH50* 50% hemolytic unit of complement, *CRP* C-reactive protein, *dsDNA antibody* Double strand deoxyribonucleic acid antibody, *eGFR* Estimated glomerular filtration rate, *HPF* High power field, *MPO-ANCA* Myeloperoxidase-antineutrophil cytoplasmic antibody, *LDH* Lactate dehydrogenase, *PR3-ANCA* Proteinase 3-antineutrophil cytoplasmic antibody

## Discussion and conclusions

Visceral disseminated VZV infection is a rare but serious complication with a high mortality rate in immunosuppressed patients such as hematological diseases or organ transplant recipients [2, 4, 6, 7, 13]. Rowland et al*.* [13] reported that visceral disseminated VZV infection occurred in 13 (4.4%) of 294 patients with acute lymphoblastic leukemia aged 0 to 15 years, and its mortality rate was 38%. Doki et al*.* [7] also reported that visceral disseminated VZV infection occurred in 20 (0.8%) of 2,411 patients who underwent allogenic stem cell transplantation, and its mortality rate was 20%. In addition, Ishikawa et al*.* [14] reported that visceral disseminated VZV infection occurred in 3,051 (10.5%) of 29,054 hospitalized patients with herpes zoster, and over 75 years of age, liver cirrhosis, heart failure and previous cerebrovascular events were poorer prognostic factors in-hospital mortality, although there are no matching factors in our case. In patients with SLE, visceral disseminated VZV infection is rare, although Habuka et al*.* [8] and Vassia et al*.* [9] recently reported the fatal cases of visceral disseminated VZV infection in patients with SLE undergoing immunosuppressive therapy consisting of PSL and MMF.

The symptoms, abdominal pain and skin blisters, and clinical course of our presented case were typical as visceral disseminated VZV infection. In most cases of visceral disseminated VZV infection, gastrointestinal symptom including abdominal pain, intestinal obstruction, vomiting and diarrhea preceded skin lesions by several days (range 4–10 days) [2]. Of gastrointestinal symptoms, the incidence of abdominal pain was 100%, although there was no specific finding in abdominal ultrasound and CT scan [2, 15–17]. The management of abdominal pain is generally difficult and requires opioid analgesics [18, 19]. The severe abdominal pain may be resulted from proliferation or inflammation by VZV in the celiac and mesenteric ganglia [19, 20], although the precise mechanism remains unclear. In addition, many patients presented moderately or profoundly elevated levels of transaminase and coagulation abnormalities [15, 18].

MMF is used as a key drug for the management of lupus nephritis [21–23], and Chan et al*.* [24] reported that MMF for diffuse proliferative lupus nephritis was more effective and associated with a lower incidence of adverse events including infections than oral cyclophosphamide. On the other hand, ALMS study cautioned that severe infection was more prevalent among Asian patients receiving MMF than those in the other regions [25]. Recently, Kwan et al*.* [26] reported that VZV infection developed in 17.1% of patients with SLE receiving MMF, but in none of patients receiving belimumab. Chakravarty et al*.* [3] also showed that patients with SLE had an increased susceptibility for VZV compared to patients with other musculoskeletal diseases, and the use of MMF was an independent risk factor for the VZV infection in patients with SLE. Furthermore, Rondaan et al*.* [27] reported that both cellular and humoral immunity to VZV weakened in patients with SLE.

Treatment for visceral disseminated VZV infection is administration of sufficient doses of acyclovir and the reduced dose of immunosuppressive drug such as MMF [3, 15]. Doki et al*.* [7] reported that most survivors were started the administration of acyclovir before or on the same day as the appearance of skin lesions, although non-survivors were started it after the appearance. Therefore, earlier diagnosis and prompt or empiric medical intervention should be needed for the management of this serious complication [16]. In our case, we actually started the administration of acyclovir and immunoglobulin on the same day as the appearance of skin lesions.

In the present case, intravenous administration of immunoglobulin (IVIG) was carried out at the onset of visceral disseminated VZV infection. Previous study has demonstrated that IVIG as replacement therapy reduced the rate of infectious events in SLE patients with secondary hypogammaglobulinemia [28]. IVIG was also recommended as one of treatment options in those patients complicated with severe infection [29, 30]. Due to the immunosuppressive therapy consisting of PSL and MMF, serum IgG level was dropped to 549 mg/dL at the onset of visceral disseminated VZV infection. In addition, we could not rule out the possibility that the patient complicated with the other infection at the onset. Therefore, we decided to carry out IVIG for the patient with hypogammaglobulinemia.

In conclusions, we presented a survival case of visceral disseminated VZV infection in a patient with SLE undergoing the immunosuppressive therapy. Our case highlights the importance of a clinical awareness of visceral disseminated VZV infection and the necessity of immediate or empiric administration of acyclovir and reduction of immunosuppressive therapy to save immunosuppressed patients.

## Data Availability

The datasets used and/or analyzed are available from the corresponding author upon reasonable request.

## Abbreviations

ACR: American College of Rheumatology
ALT: Alanine aminotransferase
AST: Aspartate aminotransferase
BUN: Blood urea nitrogen
CH50: 50% Hemolytic unit of complement
CRP: C-reactive protein
CT: Computed tomography
eGFR: Estimated glomerular filtration rate
DIC: Disseminated intravascular coagulation
DNA: Deoxyribonucleic acid
ds-DNA antibody: Double strand-deoxyribonucleic acid antibody
FDP: Fibrinogen/fibrin degradation products
HPF: High power field
IVIG: Intravenous administration of immunoglobulin
MMF: Mycophenolate mofetil
MPO-ANCA: Myeloperoxidase-antineutrophil cytoplasmic antibody
LDH: Lactate dehydrogenase
PR3-ANCA: Proteinase 3-antineutrophil cytoplasmic antibody
PSL: Prednisolone
SLE: Systemic lupus erythematosus
SELENA-SLEDAI: The Safety of Estrogens in Lupus Erythematosus National Assessment-Systemic Lupus Erythematosus Disease Activity Index
SLICC: Systemic Lupus International Collaborating Clinics
VZV: Varicella zoster virus
